# Supplementary material for: SAILER: scalable and accurate invariant representation learning for single-cell ATAC-seq processing and integration
Source: Bioinformatics. 2021 Jul 12;37(Suppl 1):i317–26. doi: 10.1093/bioinformatics/btab303 (PMC8275346; doi:10.1093/bioinformatics/btab303)
Supplement: btab303_Supplementary_Data [file btab303_supplementary_data.pdf]

Supplementary Materials for SAILER: Scalable and Accurate Invariant Representation Learning for Single-Cell ATAC-Seq Processing and Integration

Yingxin Cao[1,5,6⋆], Laiyi Fu[1,2 ⋆], Jie Wu[3], Qinke Peng[2], Qing Nie[4,5,6], Jing Zhang[1 ⋆⋆], and Xiaohui Xie[1 ⋆⋆]

[1] Department of Computer Science, University of California, Irvine, CA, 92697, USA
[2] Systems Engineering Institute, School of Electronic and Information Engineering, Xi'an Jiaotong University, Xi'an, Shannxi, 710049, China
[3] Department of Biological Chemistry, University of California, Irvine, CA, 92697, USA
[4] Department of Mathematics, University of California, Irvine, CA, 92697, USA
[5] Center for Complex Biological Systems, University of California, Irvine, CA, 92697, USA
[6] NSF-Simons Center for Multiscale Cell Fate Research, University of California, Irvine, CA, 92697, USA

⋆ Equal contributions
⋆⋆ To whom correspondence should be addressed

Table of Contents

1 Results on Hyperparameter Robustness 3
2 Derivations of the Mutual Information Objective 3
3 Results on Batch Effect Correction Benchmarking 4
4 Runtime and Scalability 4
5 Results on Batch Effect Correction on different platforms 5
6 Results on memory cost 5

List of Tables

S1 Evaluation Results under different λs. 3
S2 Evaluation results under different latent dimensions. 3
S3 Evaluation results under different intermediate neuron numbers. 3

List of Figures

S1 Clustering Performance under different Hyperparameters 6
S2 Batch effect correction comparison 7
S3 Runtime and Scalability Summaries 8
S4 Batch correction methods comparison on different sequencing platforms 9

1 Results on Hyperparameter Robustness

In this section, we present evaluation results under different hyperparameter settings. In particular, we show the mean mutual information $I(\mathbf{z}, \mathbf{c})$[1], Adjusted Rank Index (ARI), and Normalized Mutual Information (NMI) between cluster assignments and ground truth labels evaluated on the mouse atlas dataset for different hyperparameter settings. The default setting is $\lambda = 1$, $dim(\mathbf{z}) = 10$, number of intermediate neuron units is 100. For each experiment, we change one of the hyperparameter listed above. The results are shown in table S1, S2, S3 and Fig. S1.

Table S1: Evaluation Results under different λs.

λ	0	0.01	0.1	1	2	10	50
$I(\mathbf{z}, \mathbf{c})$	0.071	0.053	0.045	0.040	0.043	0.046	0.040
ARI	0.539	0.546	0.560	0.575	0.546	0.562	0.605
NMI	0.773	0.774	0.778	0.799	0.772	0.779	0.780

Table S2: Evaluation results under different latent dimensions.

$dim(\mathbf{z})$	2	5	10	15	20
$I(\mathbf{z}, \mathbf{c})$	0.105	0.059	0.040	0.038	0.0400
ARI	0.482	0.555	0.575	0.556	0.571
NMI	0.735	0.763	0.799	0.774	0.770

Table S3: Evaluation results under different intermediate neuron numbers.

# of units	50	100	200	350	500
$I(\mathbf{z}, \mathbf{c})$	0.057	0.040	0.045	0.039	0.039
ARI	0.563	0.575	0.615	0.623	0.583
NMI	0.782	0.799	0.785	0.789	0.786

2 Derivations of the Mutual Information Objective

In this section, we show the detailed derivation of the mutual information objective[2] used in our model.

With properties of Mutual Information and a variational inequality, we have

$$\begin{aligned} I(\mathbf{z}, \mathbf{c}) &= I(\mathbf{z}, \mathbf{x}) - I(\mathbf{z}, \mathbf{x}|\mathbf{c}) &(1)\\ &= I(\mathbf{z}, \mathbf{x}) - H(\mathbf{x}|\mathbf{c}) + H(\mathbf{x}|\mathbf{z}, \mathbf{c}) &(2)\\ &\leq I(\mathbf{z}, \mathbf{x}) - H(\mathbf{x}|\mathbf{c}) - \mathbb{E}_{\mathbf{x},\mathbf{c},\mathbf{z}\sim q}[\log p_\theta(\mathbf{x}|\mathbf{z}, \mathbf{c})] &(3)\\ &= \mathbb{E}_{\mathbf{x}}[D_{KL}(q_\phi(\mathbf{z}|\mathbf{x})||q_\phi(\mathbf{z}))] - H(\mathbf{x}|\mathbf{c}) - \mathbb{E}_{\mathbf{x},\mathbf{c},\mathbf{z}\sim q}[\log p_\theta(\mathbf{x}|\mathbf{z}, \mathbf{c})] &(4) \end{aligned}$$

$H(\mathbf{x}|\mathbf{c})$ doesn't involve $\mathbf{z}$, thus it could be ignored during the optimization. Terms from equation 4 looks similar as the VAE objective, with some modifications on the conditional log likelihood (equation 5).

$$L_{\text{VAE}} = \mathbb{E}_{\mathbf{x},\mathbf{c}\sim q(\mathbf{x},\mathbf{c})}\left[-\mathbb{E}_{\mathbf{z}\sim q_\phi(\mathbf{z}|\mathbf{x})}[\log p_\theta(\mathbf{x}|\mathbf{z}, \mathbf{c})] + D_{\text{KL}}(q_\phi(\mathbf{z}|\mathbf{x}) \parallel p(\mathbf{z}))\right] \quad (5)$$

According to equation 4, the ELBO of VAE is modified to minimize the negative log likelihood conditioned and the mutual information between latent variable $\mathbf{z}$ and confounding factors $\mathbf{c}$ for invariant representation learning (equation 6).

$$\min L_{\text{VAE}} + \lambda I(\mathbf{z}, \mathbf{c}) \tag{6}$$

Putting equation 4, 5 and 6 together, we have the final objective,

$$\begin{aligned} L(\phi, \theta) = \mathbb{E}_{\mathbf{x}\sim q(\mathbf{x})} \left[D_{\text{KL}}(q_\phi(\mathbf{z}|\mathbf{x}) \parallel p(\mathbf{z})) + \lambda D_{\text{KL}}(q_\phi(\mathbf{z}|\mathbf{x}) \parallel q_\phi(\mathbf{z})) \right] \\ -(1+\lambda)\mathbb{E}_{\mathbf{x},\mathbf{c}\sim q(\mathbf{x},\mathbf{c})} \left[\mathbb{E}_{\mathbf{z}\sim q_\phi(\mathbf{z}|\mathbf{x})} [\log p_\theta(\mathbf{x}|\mathbf{z}, \mathbf{c})] \right] \end{aligned} \tag{7}$$

3 Results on Batch Effect Correction Benchmarking

In this section, we show benchmark results on Sim3 dataset using SnapATAC[3] with Harmony[4] and SAILER. Sim3 dataset contains 2 batches, 6 types of cells including 2 rare cell types. Harmony is applied after dimensional reduction in SnapATAC pipeline to remove batch effect. We show UMAP visualization of latent landscape colored by cell type (Fig. S2A), t-SNE visualization of latent landscape colored by batch (Fig. S2B). As shown in the Fig. S2A, SnapATAC with Harmony failed to separate the two batch specific rare cell types (blue+orange cluster), while SAILER's unified framework separated these two cell types successfully. In Fig. S2B, SnapATAC without batch effect correction clearly shows separated batches even within the same cell type. Harmony can align different batches together, but with obvious sub-cluster patterns. On the contrary, SAILER merges these cells quite well by reporting locally homogeneous mixing from different batches.

We also calculated a quantitative measure of the mixing of cells from different batches in three steps. 1) Build the KNN graph for each cell (K=50); 2) Find the 50 nearest neighbor of each cell in the embedded space; 3) For each cell, calculate the proportion of its nearest neighbors from batch 0 and batch 1, denoted by p_0 and p_1 separately.

Intuitively, a good batch effect correction method will provide p_0 and p_1 approximately 0.5 (after cell number normalization) if two batches from different platforms is well mixed, otherwise will result in a biased mixture, as shown in Fig. S2C(a). From Fig. S2C(b), we can see that SAILER has better local mixture of two batches by reporting balanced p_1 values, as compared with SnapATAC with Harmony, which indicates improved local homogeneity, proving that our method is more robust in dealing with multiple batches.

4 Runtime and Scalability

We compared the runtime of three methods benchmarked with the mouse atlas dataset[5], namely SAILER, SCALE[6], and SnapATAC. Fig. S3A shows the runtime of three methods. Both deep learning methods SCALE and SAILER are trained thoroughly for 400 epochs using a NVIDIA RTX 2080Ti GPU. Scalability

of SAILER is tested on re-sampled mouse atlas dataset with sample size ranging from 5k to 1M. Results are shown in Fig. S3B. SAILER achieves the shortest runtime. In the meantime, runtime of SAILER scales linearly up to sample size of 1M cells in our experiment.

5 Results on Batch Effect Correction on different platforms

Last but not least, batch effects are often caused by experiments from different platforms. These platform-to-platform variations also play a vital role in separating cells apart even if they are originated from the same cell type. To evaluate the clustering performance of our method SAILER on cells from different platforms, we choose SnapATAC (with Harmony) as comparison and draw the UMAP visualization plot on two mouse brain datasets [3] generated using combinatorial indexing single nucleus ATAC-seq platform (MOs-M1/ snATAC) and droplet-based platform (Mouse Brain 10X / 10X) respectively. As the result shown in Fig. S4, both SnapATAC (with Harmony) and SAILER performs quite well in mixing these two platform cells together, further demonstrating that our model is quite robust in dealing with batch effects.

6 Results on memory cost

In terms of memory usage, since CPU based method like SnapATAC is different from GPU based methods like SAILER and SCALE, so to evenly compare its cost, we monitor the memory cost of the two GPU-based, namely SAILER and SCALE, on mouse atlas dataset during training process, their memory cost are 4319 megabytes and 4553 megabytes respectively using NVIDIA 2080ti GPU, which is just 1/3 of the maximum memory of one GPU card. Thus, the model could be expected to apply to more memory consuming datasets. The reasonable memory cost also denotes a useful application for other memory consuming datasets.

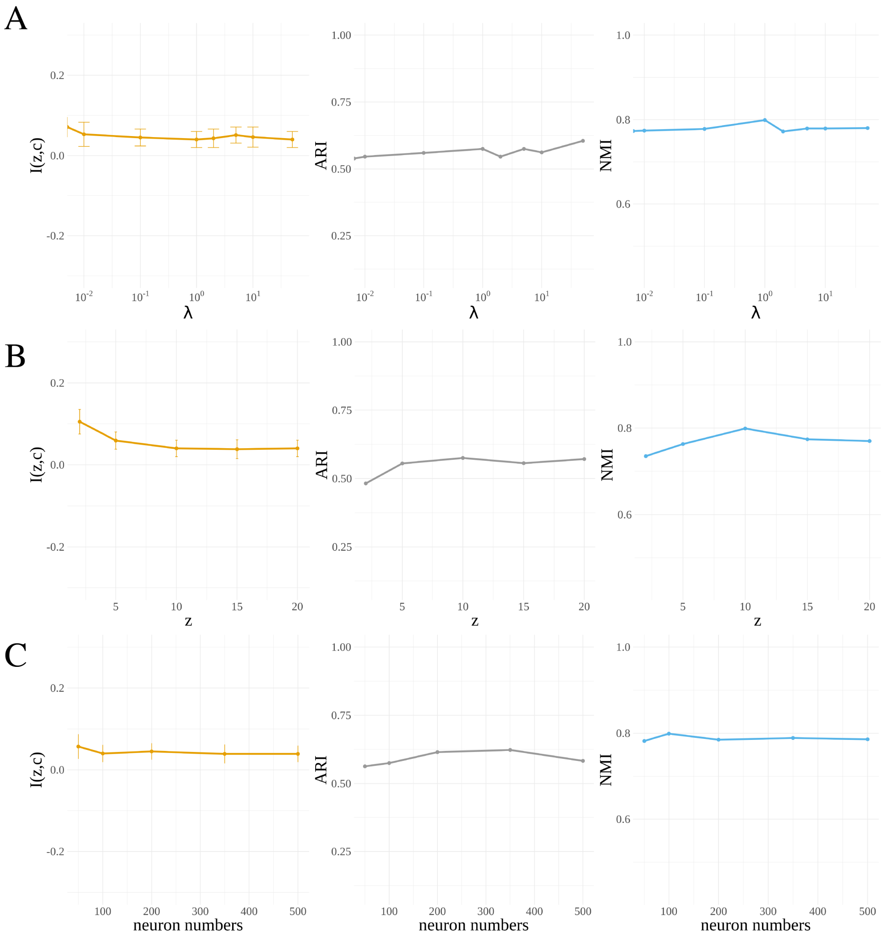

Fig. S1: Clustering Performance of $I(\mathbf{z}, \mathbf{c})$, ARI and NMI under different Hyperparameters. (A) Performance variations with λ ranging from 0.01 to 100. (B) Performance variations with latent dimension ranging from 5 to 20. (C) Performance variations using number of neurons ranging from 100 to 500.

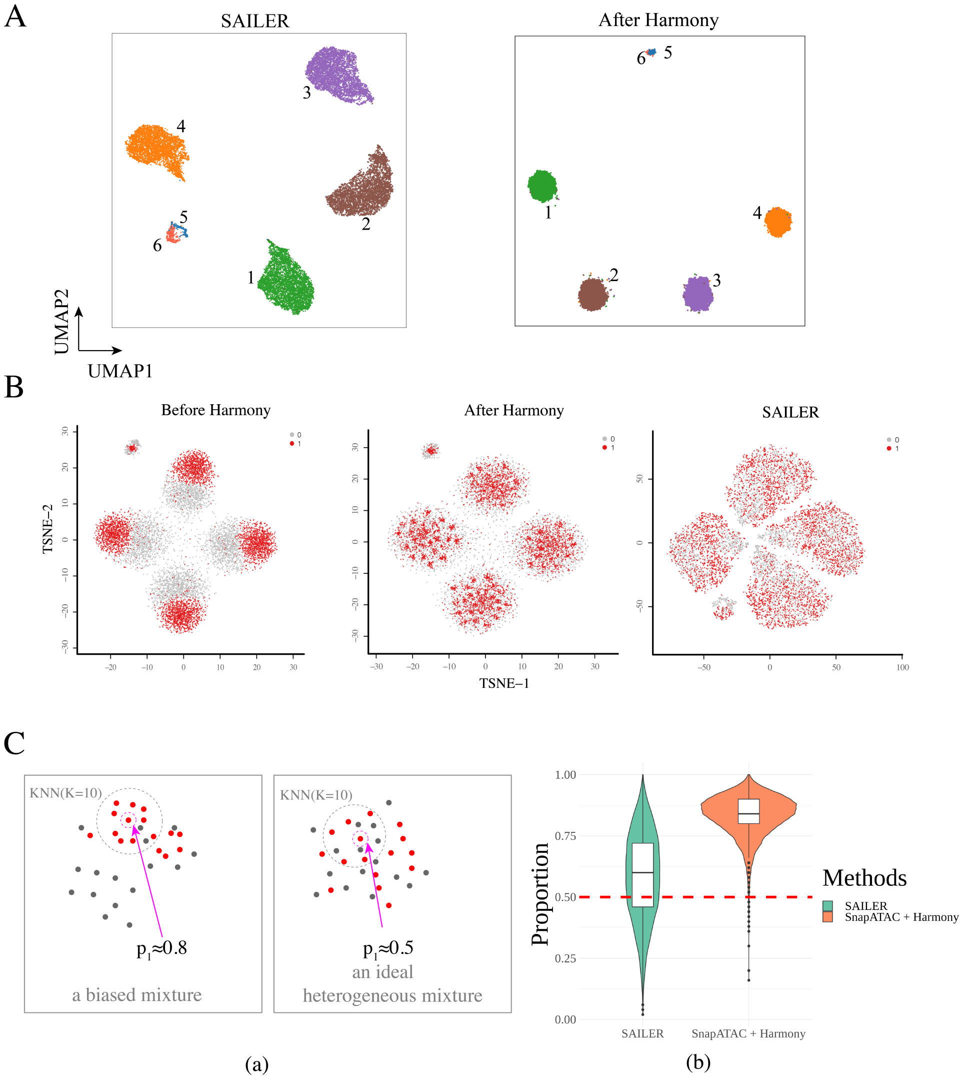

Fig. S2: Batch effect correction comparison. (A) SAILER (left) compared with other batch-effect correction SnapATAC w/ Harmony (right) on Sim3 dataset. Color indicates cell types. (B) SAILER compared with SnapATAC w/ Harmony on batch-effect correction. (C) Neighbor composition distribution for SnapATAC w/ Harmony and SAILER.

A

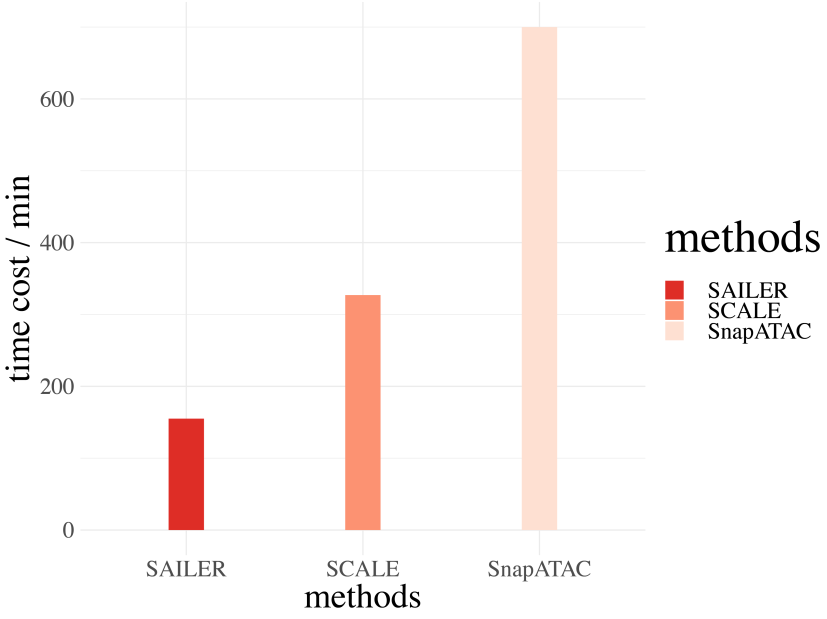

B

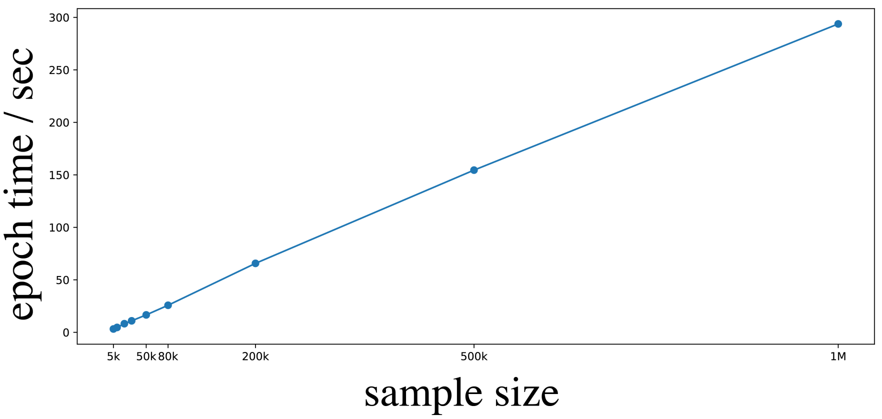

Fig. S3: Runtime and Scalability Summaries. (A) Runtime comparison of SAILER, SCALE and SnapATAC on mouse atlas dataset. (B) Scalability of SAILER for different sample sizes.

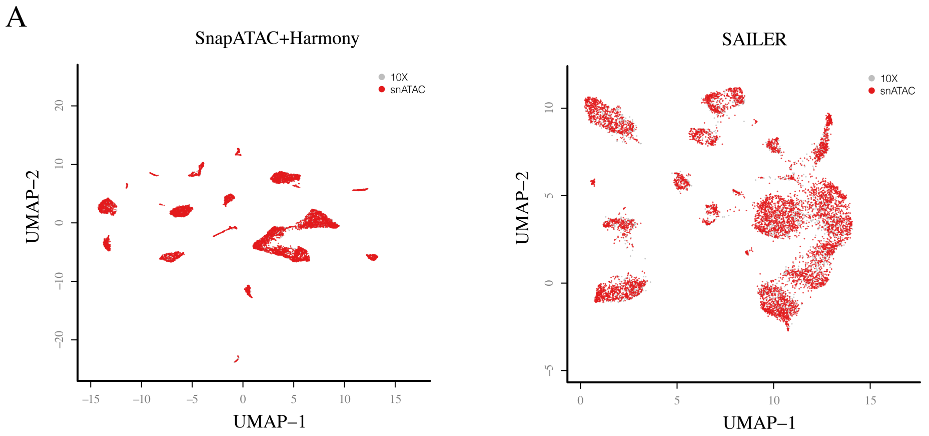

Fig. S4: Batch correction methods comparison on different sequencing platforms. (A) UMAP visualizations of latent landscapes generated by SnapATAC+Harmony (left) and SAILER (right) on merging two datasets of mouse brain generated with combinatorial indexing single nucleus ATAC-seq (MOs-M1/ snATAC) and droplet-based platform (Mouse Brain 10X / 10X) respectively.

References

1. Alexander Kraskov, Harald Stögbauer, and Peter Grassberger. Estimating mutual information. *Physical review E*, 69(6):066138, 2004.
2. Daniel Moyer, Shuyang Gao, Rob Brekelmans, Greg Ver Steeg, and Aram Galstyan. Invariant representations without adversarial training. In *Proceedings of the 32nd International Conference on Neural Information Processing Systems*, pages 9102–9111, 2018.
3. Rongxin Fang, Sebastian Preissl, Yang Li, Xiaomeng Hou, Jacinta Lucero, Xinxin Wang, Amir Motamedi, Andrew K Shiau, Xinzhu Zhou, Fangming Xie, et al. Comprehensive analysis of single cell atac-seq data with snapatac. *Nature communications*, 12(1):1–15, 2021.
4. Ilya Korsunsky, Nghia Millard, Jean Fan, Kamil Slowikowski, Fan Zhang, Kevin Wei, Yuriy Baglaenko, Michael Brenner, Po-ru Loh, and Soumya Raychaudhuri. Fast, sensitive and accurate integration of single-cell data with harmony. *Nature methods*, 16(12):1289–1296, 2019.
5. Darren A Cusanovich, Andrew J Hill, Delasa Aghamirzaie, Riza M Daza, Hannah A Pliner, Joel B Berletch, Galina N Filippova, Xingfan Huang, Lena Christiansen, William S DeWitt, et al. A single-cell atlas of in vivo mammalian chromatin accessibility. *Cell*, 174(5):1309–1324, 2018.
6. Lei Xiong, Kui Xu, Kang Tian, Yanqiu Shao, Lei Tang, Ge Gao, Michael Zhang, Tao Jiang, and Qiangfeng Cliff Zhang. Scale method for single-cell atac-seq analysis via latent feature extraction. *Nature communications*, 10(1):1–10, 2019.
